# Beyond one-size-fits-all: managing mental health calls with the integrated Behavioral Emergency Assessment and Response (iBEAR) model

**DOI:** 10.3389/fpsyg.2025.1585009

**Published:** 2025-06-10

**Authors:** Benni Zaiser, Mario S. Staller, Swen Koerner

**Affiliations:** ^1^Independent Researcher, Aurora, ON, Canada; ^2^University of Applied Sciences of Police and Public Administration North Rhine-Westphalia, Gelsenkirchen, Germany; ^3^German Sports University Cologne, Cologne, Germany

**Keywords:** behavioral emergency, person in crisis, crisis intervention, functional analysis, applied behavioral analysis, crisis response, behavioral health crisis, mental health crisis

## Abstract

Several countries have committed to improving law enforcement response to behavioral emergencies through mental health crisis response and intervention training as well as by implementing crisis intervention team programs. However, these measures often rely primarily on traditional crisis intervention methods adapted from clinical settings. At the same time, not all behavioral emergencies constitute a mental health crisis and can be responded to with a single generic approach. Psychiatric disorders, intellectual and developmental disabilities, and/or adverse life circumstances can also result in behaviors that are below crisis threshold but still prompt emergency calls. Such presentations reflect maladaptive coping mechanisms rather than a complete loss of coping capacity seen during acute crisis and include, for instance, drug-seeking criminal behaviors in individuals with substance use disorders or self-stimulatory behaviors in individuals with autism spectrum disorder, particularly when such behaviors violate social norms. Crisis intervention alone fails to fully address the complex nature of these incidents. Currently, no existing framework effectively integrates guidelines for first responders to manage both acute crises and maladaptive behaviors that do not stem from a crisis state. To fill this gap, we propose the integrated Behavioral Emergency Assessment and Response (iBEAR) model as a theory-driven framework that equips first responders with evidence-based assessment, decision-making, and response strategies, easy to access while managing potentially dynamic and stressful behavioral emergencies. The model responds to well-documented demand for enhanced training and preparedness in managing behavioral emergencies, while also addressing the increasing burden of such incidents on emergency services.

## 1 Introduction

Law enforcement has repeatedly drawn international attention for both excessive force and “lawful but awful” outcomes involving individuals experiencing mental health or behavioral crisis (CIT International, n.d.; Munetz and Bonfine, 2022; Williams et al., 2019). Recent examples include the 2024 fatal shooting of Sonya Massey in Illinois (US), who had been diagnosed with paranoid schizophrenia and called 911 during a crisis (O’Connor, 2024); Mouhamed Dramé, diagnosed with PTSD and fatally shot by police in Dortmund/Germany in 2022 after holding a knife to his throat (Fischhaber et al., 2024); and Ryan Gainer, a 15-year-old with autism spectrum disorder, killed in 2024 during a confrontation involving a gardening tool (Victoria, 2024).

Incidents like these have, in the past, led to widely mandated de-escalation and crisis intervention training, particularly in English-speaking countries where evidence-based policing has firmly established a tradition (National Police Chiefs’ Council, 2024; Office of Community Oriented Policing Services, 2015; Piza and Welsh, 2022; Public Safety Canada, 2020). Furthermore, an increasing number of jurisdictions in these countries run Crisis Intervention Team (CIT) programs, which combine specialized training for law enforcement officers in behavioral emergency response with institutionalized inter-agency collaboration for a minimally invasive, community (co-)owned behavioral crisis response (CIT International, n.d.; Munetz and Bonfine, 2022). There is also growing recognition that calls may be better served without law enforcement involvement all together (as long as they do not involve immediate safety threats). As a result, there is an increased emphasis on implementing alternative primary responders to behavioral health crises, typically involving community-based and/or (mental) health care associated clinicians and practitioners (Anene et al., 2023; Watson et al., 2025).

Research supports both crisis intervention and de-escalation training as well as CIT programs in improving law enforcement response to behavioral emergencies (Engel et al., 2022; Hassell, 2020; Watson et al., 2017). However, these approaches do not allow officers and allied behavioral health care practitioners to fully harness evidence-based best practices in behavioral crisis response. The knowledge and skills imparted in these programs focus predominantly on traditional methods of crisis intervention that originate in clinical contexts. These relate typically to working with individuals in psychiatric settings or other marginalized groups in social work contexts (cp. James and Gilliland, 2017; University of Memphis CIT Center, n.d.).

Yet, crisis intervention alone, however, does not adequately address the complexities of the wide array of behavioral emergencies that first responders^1^ encounter on a daily basis. Not all calls that are associated with a mental health crisis actually involve individuals who meet the specific criteria for a crisis state (Lorey and Fegert, 2021; Shore and Lavoie, 2019; Watson and Wood, 2017). Many of these incidents are triggered by behaviors that may appear concerning or unusual but do not necessarily constitute acute crisis (Lorey and Fegert, 2021; Shore and Lavoie, 2019; Watson and Wood, 2017). Corresponding behaviors can include, for instance, drug-seeking criminal behavior by individuals with substance use disorders, or self-stimulatory behavior (*stimming* or *self-stimulatory behavior*) by individuals diagnosed with autism spectrum disorder, where it violates social norms (cp. see Halperin et al., 1995; Perry, 2001). Since these behaviors do not necessarily stem from an acute mental health crisis, they fall outside the scope of standard crisis intervention and de-escalation training.

While CIT programs in some jurisdictions have expanded to include training beyond immediate crisis response, for instance, by incorporating content on intellectual and developmental disabilities (IDDs; e.g., Bureau of Justice Assistance, 2022; University of Memphis CIT Center, n.d.), these efforts remain largely modular and issue specific. To our knowledge, no existing model systematically integrates evidence-based strategies for both acute crisis behaviors and maladaptive behaviors below the crisis threshold in a way that is both practical and operationally effective for first responders that might be co-responding (e.g., social workers or psychiatric nurses). This gap is particularly critical in high-stakes, high-risk, and high-acuity situations, such as 911 calls involving behavioral emergencies, where elevated stress levels can interfere with cognitive functioning and hinder the recall and application of complex or abstract training content (Baldwin et al., 2022; Marlatte et al., 2025; Martin et al., 2019).

Therefore, we propose the integrated Behavioral Emergency Assessment and Response (iBEAR) model to provide first responders with a theory-driven model that integrates: (1) observed behaviors and clinically relevant background knowledge as a foundation for decision-making, (2) evidence-based intervention strategies for both behavioral crises and maladaptive behaviors below crisis threshold, and (3) situationally applicable and effective guidelines.

As such, the iBEAR model addresses a well-documented demand from first responders for enhanced training and better preparation for behavioral emergencies (e.g., Ghelani, 2022; Lorey and Fegert, 2021; Bock et al., 2015; Richmond and Gibbs, 2021; Thomas and Watson, 2017) as well as the increasing burdens associated with these types of incidents (Wittmann et al., 2020; Richmond and Gibbs, 2021; Thomas and Watson, 2017).

We start this conceptual analysis by explaining key clinical terms (section 2.). We then examine specific behaviours, along with their potential underlying psychiatric conditions, and connect them with corresponding intervention strategies. From there, we integrate these insights and devise the iBEAR model (section 3), which will assist first responders in effectively assessing and responding to behavioral emergencies. We will conclude the contribution by highlighting limitations and outlining future research and conceptual development directions (section 4).

## 2 Key concepts and scope

### 2.1 Behavioral health crisis

A behavioral or mental health crisis often refers to an acute emergency, in which psychological equilibrium is so severely disrupted that available coping mechanisms fail. This leads to general functional impairments. Such an emergency is typically a subjective response to an event, where the resulting distress exceeds an individual’s (previously acquired) coping capacity. These events are characterized by stressful, traumatic, or high-risk personal experiences (Roberts, 2000, 2005; Dross, 2001). It is important to note that each individual can experience behavioral health crisis and the corresponding loss of coping capacity at different points and experiences (Warrender et al., 2021; Sansone, 2004). Behavioral health crises have diverse causes, stemming from internal or external circumstances or a combination of both. For example, psychosis or spontaneous panic attacks can induce a crisis just as much as the loss of a loved one, a mass casualty event, overwhelming stress, or interpersonal conflicts. An underlying psychiatric condition is not required for a crisis to occur (Roberts, 2000, 2005; Sansone, 2004; Warrender et al., 2021).

### 2.2 Maladaptive behaviors below crisis threshold

Not all behaviors that indicate a behavioral health challenge and which emergency services are called to are the result of a crisis (Gou et al., 2024; Lorey and Fegert, 2021; Shore and Lavoie, 2019; Watson and Wood, 2017; Wittmann, 2022; Wittmann and Groen, 2021). As stated above, such maladaptive behaviors below crisis threshold can include drug-seeking, stimming, domestic violence but also a variety of other socially deviating and/or criminal behaviors (hence the law enforcement response). They are less the result of acute psychological destabilization but rather represent dysfunctional coping mechanisms that often create (perceived) public safety concerns, for instance drug or alcohol consumptions and associated intoxication or the experience of continued paranoid delusions. While a behavioral health crisis reflects an absence of viable coping strategies, maladaptive behaviors below crisis threshold reflect ill-adjusted but functional attempts to self-regulate and maintain an emotionally quasi-allostatic state (cp. Cortez et al., 2023; Gou et al., 2024; Wittmann, 2022; Wittmann and Groen, 2021; Zeidner and Saklofske, 1996). Reasons to involve law enforcement in such cases can include misunderstandings, conflicts that do not result in deep psychological destabilization, general concern from family or friends, or maladaptive behaviors that require intervention like, for instance, trespassing by mentally ill members of the underhoused community (Charette et al., 2011; Shore and Lavoie, 2019).

In summary, situations characterized by a loss of emotional control, such as grief following the loss of a loved one, job loss, or chronic stress, serve as examples of a mental health crisis, as discussed above (section 2.1). In contrast, maladaptive behaviors below crisis threshold include emotionally dysregulated behaviors that may not indicate an acute crisis but still require attention, such as a child’s outburst in response to parental boundary-setting or self-injurious behavior as a maladaptive coping mechanism for emotional stress.

### 2.3 Behavioral emergency

In conclusion, we define behavioral emergencies as encompassing all behavioral health challenges encountered by first responders in the law enforcement context, ranging from acute crises to maladaptive behaviors below crisis threshold (cp. Kleespies, 2017). Correspondingly, we refer to the individuals that they will encounter in their response as persons experiencing behavioral emergencies (PEBEs).

## 3 Behavioral emergencies: assessment and response

As we will demonstrate below, an acute behavioral health crisis warrants a different response from maladaptive behaviors below crisis threshold. Therefore, first responders need to categorize the type of behavioral emergency they are encountering, before taking action to respond to it. However, before they can make corresponding assessments, they have to ensure the physical safety of all involved parties (see below, section 3.1). Only then, they can engage clinically and examine the functional status of a PEBE. This status will fall into one of two categories: acute behavioral health crisis, which requires immediate crisis intervention (CI; see below, section 3.2.2), or maladaptive behaviors below crisis threshold, which require a structured behavior management (SBM) response (see below, Section 3.3). First responders will be able to assess and categorize corresponding behavioral presentations based on (a) their own observations in light of their training, education, and experience, before, during, and after their interaction with a PEBE, (b) available background knowledge provided by third parties, including family, caregivers, witnesses and 911 callers, and/or (c) local records on known presentations and diagnoses.

### 3.1 Foundation: safety

Given the inherent risks of crisis intervention during law enforcement emergency response,^2^ planning, preparing, and managing the encounter will always prioritize the safety for everyone present, including the PEBE, first responders, and others, such as family members or uninvolved third parties. This requires first responders to take measures that enable them to rapidly gain and increase control of the situation in ways that reduce risk of bodily harm to anybody involved.^3^ If immediate control over the person experiencing the behavioral emergency is not possible, for instance, due to elevated risk associated with a weapon, the approach will involve isolation and containment of the subject, securing a perimeter to ensure scene control and public safety, and establishing communications for behavioral management at the earliest possible time (cp. McMains et al., 2020; Slatkin, 2015).

An armed individual experiencing acute psychosis exemplifies such a scenario. In these situations, the priority is to ensure the safety of others before isolating and physically containing the PEBE, such as securing them in a room or a house (“isolate and contain”). Further examples that require immediate action to ensure safety include (but are not limited to) suicide attempts in progress (e.g., running into traffic or an attempted drug overdose), non-suicidal self-injuring (NSSI) behaviors with a lethal risk (e.g., self-harm under the influence of alcohol or drug overdose; for more details, see below, section 3.3.1.2), dangerous behaviors posing a lethal risk to others (e.g., an active homicide attempt or uncontrolled violent assault), or dangerous behaviors posing a non-lethal risk to others (e.g., physical assault without a weapon or verbal threats).

If first responders can ensure safety through (a) direct intervention (e.g., hands-on physical control), (b) indirect intervention (e.g., controlling and designing the immediate environment), or (c) without active intervention, they can then proceed to de-escalate the situation and prevent further physical confrontation or re-escalation (Dunham et al., 2020; Zaiser et al., 2023). This, in turn, allows for further support of the PEBE and their stabilization.

### 3.2 Behavioral emergency category: behavioral health crisis

If the PEBE is in a state of acute behavioral health crisis, first responders can implement CI strategies to counteract the individual’s sense of loss of control and help them regain and strengthen their ability to cope (Roberts, 2000, 2005; Dross, 2001). Immediate CI is crucial when behaviors emerge that pose an imminent risk of harm to the PEBE or others present, or when the situation demands an urgent response (see James and Gilliland, 2017; Roberts, 2000, 2005; Slaikeu, 1990).

These behaviors often occur alongside or as manifestations of tendencies associated with specific mental disorders^4^ and will be discussed in detail below (James and Gilliland, 2017; Roberts, 2000, 2005).

#### 3.2.1 Assessment

##### 3.2.1.1 Suicidal behaviors

Suicidal behaviors include the presence of suicidal thoughts as well as low-risk/non-viable suicidal self-injury, such as low quantity or low potency medication overdoses. It is important to distinguish suicidal self-injuring (SSI) from NSSI behaviors (Grandclerc et al., 2016; Hamza et al., 2012, for more details, see below, section 3.3.1.2). Furthermore, suicidal thoughts and behaviors can be symptoms of a wide range of mental disorders, including, among others, major depressive disorders, bipolar and related disorders, trauma- and stress-related disorders, psychotic disorders, or borderline personality disorder (BPD). However, the presence of a mental disorder does not inherently indicate suicidality. Likewise, individuals without mental illness or a psychiatric diagnosis may also exhibit suicidal behaviors.

##### 3.2.1.2 Psychotic behaviors

Psychosis is characterized by a loss of contact with reality and is often associated with various mental disorders. Observable symptoms for first responders include hallucinations (i.e., perceptions that occur without external stimuli/perceiving stimuli that are not real; American Psychological Association, 2013), delusions (i.e., fixed beliefs based on incorrect inferences about external reality, despite clear evidence to the contrary; American Psychological Association, 2013), disorganized thinking and speech, as well as potentially disorganized or abnormal motor behavior. Disorders in which such behaviors may be present include, among others, schizophrenia spectrum disorders, bipolar and related disorders, delusional disorder, substance−/medication-induced psychotic disorder, or psychotic episodes due to a physiological condition, such as delusions and hallucinations following urinary tract infections in elderly people (cp. Mostafa and Miller, 2014).

##### 3.2.1.3 Other behaviors that significantly interfere with the ability to function

Behavioral tendencies and symptoms of a variety of further psychiatric disorders as well as singular or cumulative adverse life events can also interfere with PEBEs’ ability to function, often to the extent that they lose their coping capacity and enter a state of crisis. This can occur, for instance, due to trauma- and stress-related disorders, anxiety disorders (such as panic disorder), catatonia (characterized by motor immobility, excessive motor activity, or peculiar voluntary movements). Also, initially maladaptive behavioral tendencies below crisis threshold associated with the aforementioned psychiatric disorders, which do not reflect suicidal or psychotic behavior, such as depressive disorders, bipolar and related disorders, or substance use disorders, might cause a PEBE to experience a behavioral health crisis.

#### 3.2.2 Response: crisis intervention

Suicidal and psychotic as well as other behaviors that significantly interfere with the ability to function are best responded to using immediate CI.^5^ These are rooted in general crisis intervention literature and tailored to the law enforcement context, for instance, in the Crisis Response and Intervention Training (CRIT) curriculum of the United States Bureau of Justice Assistance (2022). In general, CI aims at restoring the coping ability of the PEBE by (a) continually assessing risk, (b) providing emotional stabilization through de-escalation, (c) facilitating coping strategies through collaborative problem-solving, (d) facilitating an outcome by ensuring appropriate resource referral, and (e) completing effective follow-up (Roberts, 2000, 2005; Dross, 2001). The following sub-sections will provide a brief overview for each CI element in corresponding order, with the exception of follow-up, which lies outside the temporal scope of the initial behavioral emergency and may often not be completed by first responders. The relevant literature offers a wide range of theory- and evidence-based models that provide concrete guidance for effective CI approaches (for CI in general, see, for instance, Aguilera, 1998; James and Gilliland, 2017; Kleespies, 2017; Myer et al., 2013; Roberts, 2000, 2005; for CI in law enforcement contexts, see, for instance, CIT International, n.d.; Police Executive Research Forum, 2016; University of Memphis CIT Center, n.d.; Vecchi et al., 2005, 2019).

##### 3.2.2.1 Risk assessment

Risk assessment overlaps with ensuring overall safety (as discussed above in section 3.1) but is wider in scope, as it reaches beyond immediate safety risks during initial engagement. Once safety is established and first responders engage a PEBE with CI, they have to continually assess a variety of risks to the PEBE themselves (e.g., suicide or non-suicidal self-harm risk or risk of failure to take care of themselves) and/or to others (e.g., homicidal ideation, non-homicidal harm to others like, for instance, noxious substances administered as a result of paranoid delusions). This mitigates their materialization during or after the encounter and informs further decision making regarding hospitalization and/or resource referral (cp. Aguilera, 1998; James and Gilliland, 2017; Kleespies, 2017; Myer et al., 2013; Roberts, 2000, 2005). Steps to assess risk include the recognition of warning signs, such as looking for expressions of hopelessness, helplessness, and worthlessness in suicidal PEBEs or fixation on others in context of retaliation, anger, or disappointment in PEBEs, where there is a concern of harm to others. To assess such risk more systematically, the CRIT curriculum proposes the S. A. L. method (Bureau of Justice Assistance, 2022):

- Specificity of the plan to suicide/harm themselves or others,
- Availability/accessibility of the means to carry out the plan,
- Lethality/viability of the method.

##### 3.2.2.2 De-escalation and stabilization

The Behavioral Influence Stairway Model (BISM; Vecchi et al., 2005, 2019) provides a simple, three-step process to de-escalate, stabilize, and positively influence a PEBE. Originally an adaption of conventional CI principles to police crisis negotiations, the BISM is now increasingly taught to general duty police officers as an effective aid to train and practice de-escalation in the field (e.g., Bureau of Justice Assistance, 2022; Barath, 2023). First responders have to first empathize with the PEBE. This affords progression to the second step and the building of rapport. Once they have built rapport with the subject, they can proceed to the third step and influence the PEBE towards facilitating coping strategies through collaborative problem-solving, accepting referral to an appropriate resource (e.g., health care facility, community-based services, or family, friends, or other caregivers). Throughout this three-step process, first responders need to listen actively (for an overview of active listening skills, see Zaiser and Staller, 2015), as they shift from a control-oriented to an influence-oriented mindset, offering small, respectful choices rather than issuing commands: “Using a less authoritarian, less commanding and less confrontational approach, can give you more control” (Bureau of Justice Assistance, 2022, Slide 16.5).

##### 3.2.2.3 Problem-solving

Once there is a viable level of rapport established, first responders can gradually move toward behavioral influence and engage the PEBE in collaborative problem-solving. Maintaining unconditional PEBE-centricity (Rogers, 1940, 1959), they can frame them not as a passive recipient of intervention but as an active participant in restoring their own coping capacity. First responders can guide them to recall strategies that have previously helped them regain stability (e.g., contacting a support person, using grounding techniques, relocating to a calmer environment) or collaboratively explore new coping actions that the PEBE can implement independently or with minimal support. Examples include helping the PEBE articulate what environment feels safest to them in that moment, encouraging them to take deep breaths if previously found helpful, or offering simple, non-coercive choices such as sitting in a quieter area or contacting a trusted family member (e.g., James and Gilliland, 2017; Roberts, 2000, 2005). By presenting pathways rather than demands, first responders foster collaboration, reduce power struggles, and increase the PEBE’s engagement in co-creating a solution. Imposing solutions like involuntary psychiatric holds and or hospitalization have to remain the last resort (e.g., Bureau of Justice Assistance, 2022; James and Gilliland, 2017; Roberts, 2000, 2005).

##### 3.2.2.4 Referral

Once the PEBE is stabilizing, first responders should work toward facilitating an appropriate referral to external resources for additional help in restoring coping capacity and/or ensuring sustainability. Referral decisions must be based on ongoing risk assessment, the PEBE’s stated needs and preferences, and the principle of using the least restrictive alternative consistent with safety (Roberts, 2005; James and Gilliland, 2017; Myer et al., 2013). Referral is an extension of the collaborative problem-solving process, maintaining respect for the PEBE’s autonomy whenever feasible. First responders will benefit from consulting with available CIT officers, co-response teams, or on-call clinicians to ensure the referral decision is clinically informed and aligned with available community resources. If high risk persists and voluntary cooperation is not possible, involuntary hospitalization must be pursued only after less restrictive alternatives have been ruled out and with full respect for the PEBE’s dignity.

### 3.3 Behavioral emergency category: maladaptive behaviors below crisis threshold

If PEBEs are in a state that is characterized by maladaptive behaviors below crisis threshold, have not completely lost control, and/or are displaying behaviors that require monitoring and management beyond the initial encounter, first responders can apply SBM to address the maladaptive behaviors that led to the emergency call. Through collaboration with the PEBE and their caregivers or support system, first responders can help pave the way toward more adaptive behaviors (James and Gilliland, 2017; Roberts, 2000, 2005; Slaikeu, 1990). PEBEs may also find themselves in this exceptional state immediately following a mental health crisis, requiring structured support to stabilize and regulate subsequent behaviors. Behavioral presentations and associated disorders responsive to SBM are discussed subsequently before we provide pointers for SBM measures.

#### 3.3.1 Assessment

##### 3.3.1.1 Instrumental suicidal behaviors

Threatening suicide is a complex behavior that is not always tied to a genuine intent to die. It may instead serve instrumental purposes, commonly referred to as instrumental suicidal behavior or suicidal gestures, used to achieve certain goals (Del Bello et al., 2015; García-Nieto et al., 2014; Heilbron et al., 2010; Kessler et al., 2005). While all suicide threats must be taken seriously, structured risk assessments can help identify underlying motives such as unmet emotional or social needs, including seeking support, influencing the environment, or eliciting emotional responses from others (Nock and Prinstein, 2004; Robinson et al., 2023; Syme and Hagen, 2019; May and Klonsky, 2013; Nock et al., 2008; Wedig et al., 2013).

Complicating matters further, suicidal intent is rarely binary: self-injurious behavior exists along a continuum, from sub-intentional harm, which can include NSSI (see below, section 3.3.1.2), to fully intentional suicide attempts (Harriss et al., 2005; Kovacs and Beck, 1977; Cholbi, 2007; Lester, 2009). Since habitual instrumental suicidal behaviors often overlap with actual suicidal behavior (Robinson et al., 2023) and first responders can never definitively determine a person’s true intent, instrumental suicidal behavior must always be taken seriously and fully assessed, even when intent appears ambiguous. In addition, it can also carry a high risk of unintended harm (Crump et al., 2013; Neeleman, 2001). For instance, a PEBE may unintentionally cause life-threatening injuries through NSSI, or suffer fatal consequences when attempting to stage a suicide by stepping into traffic.

When instrumental suicidal behavior reflects maladaptive coping strategies rather than acute behavioral crisis, it will benefit much more from SBM than from CI. Psychiatric disorders commonly associated with instrumental suicidal behavior include, but are not limited to, antisocial, histrionic, and BPD, as well as neurodevelopmental disorders such as autism spectrum disorder, attention-deficit/hyperactivity disorder (ADHD), and intellectual developmental disorders.

##### 3.3.1.2 Non-suicidal self-endangering behaviors

Non-suicidal self-endangering behavior can be understood as either indirectly or directly harmful actions (Nock, 2010). Indirectly harmful behaviors include (a) compromises, where harm is knowingly accepted for a perceived benefit (e.g., alcohol or tobacco use), (b) counterproductive strategies, where goal-directed behavior unintentionally leads to harm (e.g., procrastination, learned helplessness; Baumeister and Scher, 1988; Steel, 2007), and (c) risk-taking behaviors, where engaging in high-risk activities is preferred over low-risk alternatives (e.g., reckless driving, drug overdose; Nock, 2010). Directly harmful behaviors include (a) suicidal acts, (b) instrumental suicidal behavior, and (c) NSSI, the latter defined as deliberate self-inflicted harm without suicidal intent (Cipriano et al., 2017; Nock, 2010). Common forms of NSSI are cutting, burning, or hitting oneself (Claes and Vandereycken, 2007; Whitlock et al., 2008). Self-injury typically serves two primary functions (Nock, 2010; Nock and Prinstein, 2004): cognitive or emotional regulation by reducing distress or enhancing a sense of control, or social regulation by seeking support or avoiding unwanted social situations.

Such behaviors are commonly observed across a range of psychiatric disorders. In borderline personality disorder, NSSI is often used for emotional regulation, relief from dissociation, self-punishment, expression of internal pain, or to influence interpersonal relationships. Individuals experiencing a major depressive episode may engage in non-suicidal self-endangering behavior as a coping mechanism to manage intense emotional pain or numbness (Claes and Vandereycken, 2007; Nock and Prinstein, 2004). Though less common, individuals diagnosed with autism spectrum disorder (ASD) may also engage in self-injury, often in response to sensory overload, frustration, or difficulties expressing their needs and emotions (Moseley et al., 2020).

##### 3.3.1.3 Other aggressive and disruptive behaviors

Finally, certain behavioral tendencies and symptoms associated with a range of psychiatric disorders can contribute to aggressive, disruptive, or otherwise socially inappropriate behavior. Neurodevelopmental disorders (including ASD) and intellectual and developmental disabilities (IDDs) may involve aggression, impulsivity, rigidity, sensory hypersensitivity, inappropriate social behavior, and communication difficulties, which can lead to frustration and reactive aggression (Visser et al., 2014). Neurodegenerative disorders, such as Alzheimer’s, Parkinson’s, and Huntington’s disease, are also associated with symptoms like irritability, impulsivity, wandering, and socially inappropriate actions, including sexually intrusive behavior. Personality disorders (including borderline, antisocial, and paranoid types) can involve disregard for social norms, deceitful or reckless behavior, impulsivity, hostility, excessive emotionality, and physical aggression (Swearer et al., 1988). Similarly, disruptive and impulse control disorders, such as oppositional defiant disorder (ODD) and conduct disorder, may present with aggression toward people or animals, defiance of authority, repeated rule-breaking, and deliberate provocation.

#### 3.3.2 Response: structured behavior management (SBM)

Once safety is ensured (cp. section 3.1) and immediate CI provided, if needed (cp. section 3.2), first responders can support PEBEs with SBM to address recurring maladaptive behaviors. Unlike CI, which focuses on short-term emotional stabilization, SBM aims to reshape behavior over time by managing environmental triggers and applying planned reinforcement strategies. It is rooted in behaviorist principles, which view behavior as shaped either by reflexive responses to environmental stimuli or by individual learning experiences, particularly through patterns of reinforcement and negative consequences (Watson, 2017). In respondent conditioning (also known as classical conditioning), behaviors are automatically elicited when previously neutral stimuli become associated with biologically significant events, leading to conditioned responses over time (Pavlov, 1927; Bouton, 2007). An example would be a PEBE becoming visibly anxious at the sight of a police uniform after a past coercive encounter or freezing when a door slams, due to a history of domestic violence (Pavlov, 1927; Bouton, 2007). Beyond reflexive responses, operant conditioning, a foundational concept in learning psychology, emphasizes how behaviors are strengthened or weakened based on their consequences: positive or negative contingencies that either reinforce adaptive actions or reduce maladaptive ones (Skinner, 1938; Staddon, 2016). For instance, a PEBE may continue making threats if it consistently results in being left alone, or may learn to request a break calmly if that reliably leads to reduced demands.

To operationalize these principles, SBM incorporates Functional Analysis (FA) and Applied Behavior Analysis (ABA).^6^ FA identifies the function of behavior by analyzing its ABCs: the antecedents, the behavior itself, and its consequences (Skinner, 1938). For example, a PEBE’s aggressive behavior may be reinforced by the removal of overstimulating demands (negative reinforcement) or by attention from first responders (positive reinforcement), both of which can unintentionally increase the likelihood of future aggression in similar contexts. ABA then builds on these insights to implement structured, evidence-based interventions that reduce maladaptive behavior and reinforce safer alternatives (Carr and Leblanc, 2003; Melanson and Fahmie, 2023; Cook et al., 2012; Pierce and Cheney, 2013; Rummel et al., 2012; Yoman, 2008).

This evidence-based approach has been widely applied and studied across various populations, including individuals with neurodevelopmental disorders such as ASD (Fisher et al., 2021; Yu et al., 2020; Reichow et al., 2012), IDDs (Hassiotis et al., 2011; Ho et al., 2021), and ADHD (Coles et al., 2005; Daley et al., 2014). In addition, ABA principles have been adapted for neurodegenerative disorders such as dementia (Buchanan, 2006; Panerai, 2021), conduct disorder and ODD (Drugli et al., 2010; Ghosh et al., 2017), as well as for managing specific behaviors associated with personality disorders like BPD (Linehan and Wilks, 2015; Waltz and Linehan, 1999) and mood disorders, such as bipolar disorder (Matthews-Carr, 2017).

Consequently, we propose SBM to represent the practical adaptation of ABA principles for use by first responders during behavioral emergencies. The following section outlines corresponding, specific strategies and provides guidance on their effective application.

##### 3.3.2.1 General considerations

The following SBM strategies are best delivered using a patient, structured, and transparent approach (Cooper et al., 2019; Bureau of Justice Assistance, 2022; Fisher et al., 2021; Marquenie et al., 2011). First responders should focus on (a) setting the stage for success, (b) adjusting their communication style, and (c) responding calmly to behavioral challenges as they arise (Bureau of Justice Assistance, 2022).

To set the stage and prepare a supportive environment, first responders should begin by gathering critical information from individuals familiar with the PEBE, such as family members, caregivers, social workers, or the person who initiated the emergency call. This information may include the PEBE’s daily routines, typical schedule, preferences, and known triggers or sensitivities. The interaction should ideally take place in a quiet, controlled space free from unnecessary distractions. First responders should clearly identify themselves and explain their role in simple, accessible terms.

During initial communication, plain and simple language should be used, along with short, direct sentences. Instructions should be broken down into manageable parts, with one clear step offered at a time. To build trust and reduce anxiety, first responders should explain their actions before carrying them out and avoid physical contact unless absolutely necessary for safety or explicitly welcomed by the PEBE. It is also essential that officers never lie or intentionally mislead the person, as even seemingly harmless deceptions can damage rapport and increase distress.

When facing challenges such as distractibility, emotional dysregulation, or aggressive behavior, patience is key. Where appropriate, officers can offer alternative stress-reduction options, such as inviting the individual to draw, write, or engage in a low-demand physical task like walking while talking. In cases of non-compliance or oppositional behavior, a stance of firm but calm persistence is more effective than escalation. Finally, all stakeholders involved, including all first responders as well as caregivers, should maintain consistent messaging to avoid “splitting” behaviors, where the individual attempts to exploit perceived contradictions and inconsistencies in response strategies between different first responders.

##### 3.3.2.2 Functional communication prompting

Functional communication prompting encourages the PEBE to express needs or emotions using safer, alternative forms of communication (Carr and Leblanc, 2003; Fisher et al., 2021). For instance, when a PEBE clenches their fists and looks agitated, the first responder might prompt “you can tell me if you need space, no need to get upset.” Alternatively, if a PEBE begins pacing aggressively, the officer can offer a simple prompt like “if you need to step outside to calm down, just say so,” providing a verbal outlet before escalation occurs.

##### 3.3.2.3 Differential reinforcement

Differential reinforcement involves immediately reinforcing safer, calmer behaviors while minimizing attention to maladaptive behaviors such as aggression or defiance (Cooper et al., 2019; Pierce and Cheney, 2013; Thompson and Iwata, 2005). For instance, if a PEBE initially shouts threats but then calmly asks for a chair, the first responder can acknowledge the calm behavior without revisiting the earlier aggression. Similarly, if a PEBE stops pacing and begins speaking clearly, the first responder can reinforce the calm tone by engaging positively, for instance saying: “I can hear you better now, let us keep talking like this.” This approach encourages the repetition of safe, adaptive behaviors during the encounter.

##### 3.3.2.4 Shaping small successes

Shaping small successes involves breaking larger tasks into smaller, achievable steps and reinforcing incremental progress (Cooper et al., 2019; Rummel et al., 2012). For instance, when asking a PEBE to move away from a crowded area, the first responder might first request taking two steps back: “Good, just a little back, that’s perfect,” before proceeding to a full relocation. Similarly, if a PEBE resists entering a police vehicle for transport to a hospital, the officer might first shape compliance by reinforcing the act of calmly standing by the open door, saying “you are doing great, standing here is the first step,” before encouraging entry.

##### 3.3.2.5 Setting limits/boundaries and enforcing consequences

Setting clear behavioral limits/boundaries and calmly enforcing consequences when those limits are breached is essential to maintaining safety and structure (Aiyegbusi and Kelly, 2012; Cooper et al., 2019; Didden et al., 1997). For example, the first responder might state: “You can stay here and talk with me, but if you continue throwing objects, we will need to move you to a safer area,” giving the PEBE clear expectations and associated outcomes. Similarly, if a PEBE repeatedly tries to move into traffic, the first responder might say: “If you walk into the road again, we will have to physically guide you away for your safety,” and consistently follow through if necessary. Likewise, emergency services will benefit from respectfully yet immediately and assertively explaining proper procedure to a PEBE, who requests a certain type of response or a specific first responder and refuse to cooperate with others. Predictable, consistently applied limits help reduce escalation by clarifying expectations early.

##### 3.3.2.6 Managing reinforcement contingencies

Managing reinforcement contingencies means controlling when and how reinforcement is delivered to avoid unintentionally reinforcing unsafe behaviors (Melanson and Fahmie, 2023; Petry, 2011; Yoman, 2008; Thompson and Iwata, 2005). For instance, if a PEBE demands to leave aggressively, the first responder refrains from granting the request immediately and instead focuses on calming dialogue, along the lines of “we need to calm first, then we can work on leaving.” Similarly, if a PEBE uses hostile language to demand water, the first responder can wait until the PEBE uses calmer communication before providing it, ensuring that respectful requests are reinforced.

##### 3.3.2.7 Environmental adjustments

Environmental adjustments involve modifying surroundings to reduce external triggers and support behavioral stabilization (Cook et al., 2012; Buchanan, 2006). For instance, if a PEBE becomes increasingly agitated by flashing lights of emergency vehicles, first responders might move the conversation indoors or around a corner to minimize stimulation. Similarly, if a PEBE appears overwhelmed, excited, triggered, or encouraged to communicate aggressively or inappropriately by a gathering audience, first responders can reduce the number of responding units and people present during the encounter or relocate the individual to a quieter, more private area to facilitate de-escalation.

##### 3.3.2.8 Special consideration: frequent service users

These strategies also offer an effective framework for managing protracted and frequent service users by providing structured and repeatable approaches that can shape safer, more adaptive behaviors over time. Differential reinforcement shifts attention to calm, appropriate behaviors; functional communication prompting builds alternative ways to express needs; and shaping small successes encourages gradual cooperation. Managing reinforcement contingencies prevents unintentional rewards for aggressive or attention-seeking behavior, while environmental adjustments help reduce recurring triggers. Consistent messaging across all stakeholders (all first responders, health care providers, and family members) is equally critical to prevent “splitting” behaviors, where a PEBE individuals, often in the context of emotional dysregulation (e.g., as a BPD symptom), attempts to capitalize on inconsistencies to reinforce maladaptive relational patterns (Kernberg, 1975; Linehan, 1993). For instance, when managing a frequent service user with cognitive impairment who often escalates during welfare checks, first responders might reinforce calm conversation, prompt simple verbal requests, praise small cooperative actions, avoid reacting to outbursts, reduce environmental triggers, and coordinate consistent expectations with caregivers. Over time, these strategies can help reduce service calls, increase voluntary cooperation, and create safer, more stable interactions.

An example illustrating FA and ABA-based SBM involves a teenager diagnosed with an anxiety disorder and conduct disorder. This comorbidity is well-documented in the literature (Lahey and Waldman, 2003; Moffitt et al., 2001) and associated with the hypothesis that aggressive behavior may function as a coping mechanism for underlying anxiety (Frick and Morris, 2004; Lindner et al., 2018). For instance, verbal aggression and property damage might be a maladaptive strategy used by the PEBE to avoid going to school due to fear of social exclusion and emotional distress. When first responders arrive, they can gather information from parents and social workers to identify a behavioral pattern (as per FA). Suppose they determine that the PEBE has successfully avoided school multiple times in the past through aggressive outbursts. Using ABA principles, first responders can then evaluate the potential consequences of their intervention: On the one hand, if they transfer the PEBE to a psychiatric facility, the maladaptive behavior might be positively reinforced, as it allows the individual to escape school. On the other hand, this transfer might also unintentionally reinforce aggressive outbursts, especially if it is the PEBE’s first time in a psychiatric setting, where increased stress could exacerbate symptoms of both the anxiety disorder and conduct disorder. Conversely, a collaborative approach that involves the PEBE, their family, and professionals, which may work out a way to keep them in their familiar structure and routine, has the potential to positively reinforce more adaptive behaviors and ultimately fostering long-term behavior change.

For a deeper understanding of SBM, first responders are encouraged to consult the literature on FA and ABA, which provide structured frameworks for systematically assessing behavioral functions and implementing strategic interventions (Carr and Leblanc, 2003; Melanson and Fahmie, 2023; Cook et al., 2012; Fisher et al., 2021; Behavior Analyst Certification Board, 2024; Yu et al., 2020; Reichow et al., 2012; Cooper et al., 2019; Dillenburger and Keenan, 2009).

### 3.4 Integrated behavioral emergency assessment and response

**Figure 1:**
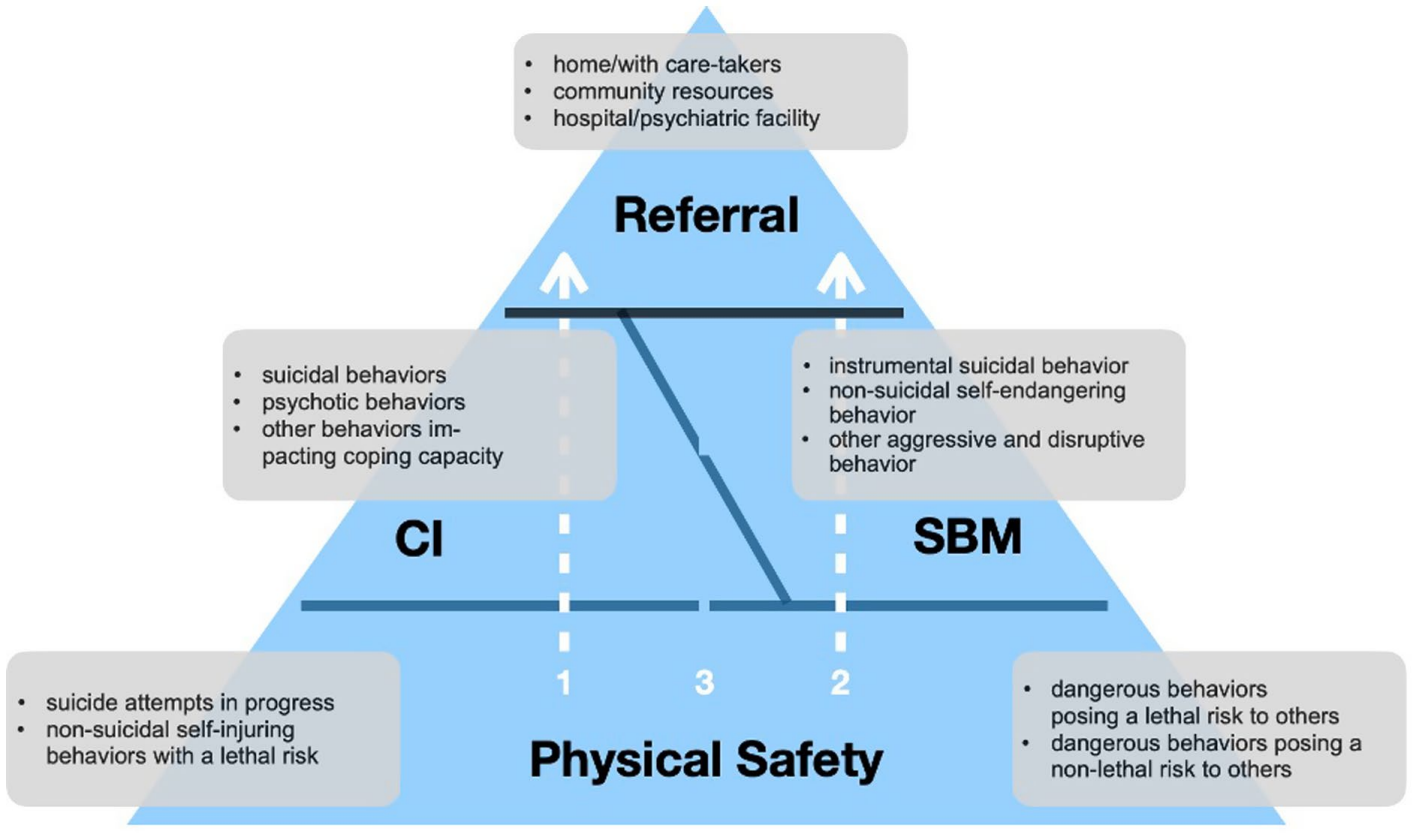
Schematic representation of the iBEAR model.

Figure 1 integrates the considerations laid out and illustrates a pathway to the effective management of behavioral emergencies. On the foundation of ensuring the physical safety of all involved parties, first responders can, contingent on the presentation of the PEBE, engage in immediate CI (arrow 1), SBM (arrow 2), or combine the two, with initial CI being followed by SBM (arrow 3). Ultimately, they transfer them either to acute psychiatric care or back to their social environment.

A scenario that illustrates the integration of CI and SBM (see Figure 1, arrow 3) involves an emergency call in which first responders are dispatched to assist a teenager diagnosed with BPD during an acute behavioral health crisis. After an emotionally charged argument with their parents, the PEBE has locked themselves in their room, made suicidal threats, and cut their arm with a piece of broken glass. These behaviours may reflect a maladaptive coping response, instrumental suicidal intent, or actual suicidal intent. The parents, alarmed, contact emergency services.

Upon arrival, first responders begin by addressing immediate safety risks, the foundational level of the iBEAR pyramid, and remove the parents from the area, securing the hallway, and containing the scene to prevent further harm. Due to the PEBE refusing to unlock the bathroom and to surrender the glass, first responders might create a situation that would require them to use of force by entering the room in a premature attempt to resolve the situation (cp. officer-created jeopardy).^7^

At this point, everyone on scene, including the PEBE, can be considered safer with containment than following police confrontation. This stability creates the conditions for CI to begin. Using calm, non-threatening verbal engagement through the closed door, first responders aim to de-escalate the situation, following the BISM (see above, section 3.2.2.2), and start to problem-solve by helping recruit proven coping strategies from the past (see above, section 3.2.2.3). The goal is to help the PEBE regain emotional control, reduce the immediate risk of further self-injury, and establish the groundwork for voluntary cooperation.

Once a basic level of rapport is established and the PEBE shows signs of emotional regulation, officers gradually transition from CI to SBM by reinforcing small behavioral successes, such as unlocking the door, stepping into the hallway, surrendering the glass, or attempting a previously discussed coping strategy, even if the individual is not yet fully regulated (see section 3.3.2.4). Upon confirming that the self-inflicted injuries are superficial and no longer bleeding, the scene no longer poses an acute threat, enabling a full shift to SBM. All attention now turns to the behavioral dynamics that contributed to the crisis.

For instance, the PEBE may have learned that self-harming behaviors and suicidal threats reliably elicit intense responses from family, police, and paramedics, serving as a means of expressing unmet emotional needs, such as attention and connection or regaining a sense of control (Agrawal et al., 2004; Torrico et al., 2024). The attention received from these responders may function as behavioral reinforcement, increasing the likelihood of similar behaviors in the future, as the PEBE comes to associate such actions with a guaranteed emergency response. To interrupt this reinforcement cycle and prevent it from becoming entrenched, responders can apply several SBM strategies. They begin by prompting functional communication (see above, section 3.3.2.2) by encouraging the PEBE to express distress or needs verbally rather than through self-harm. Calm, cooperative behaviors, such as verbalizing emotions or complying with simple requests, are then differentially reinforced (section 3.3.2.3) through supportive interaction, while escalation or self-injury is met with only essential safety interventions. First responders also reinforce incremental steps, even if partial, to build behavioral momentum shaping small successes, see above, section 3.3.2.4. Simultaneously, clear and respectful limits are set (see above, section 3.3.2.5) to reinforce personal agency and structure, for example, by explaining that additional support will only be called if safety deteriorates, while calm engagement allows the PEBE to retain more autonomy. To avoid reinforcing the crisis behavior itself, first responders carefully manage reinforcement contingencies (see above, section 3.3.2.6) by gradually withdrawing their physical presence and attention as stability increases, ensuring that positive engagement (not escalation) drives continued support. Environmental adjustments (see above, section 3.3.2.7) further support regulation by minimizing stimuli, such as removing unnecessary personnel and creating a quieter, more controlled setting.

Finally, rather than defaulting to psychiatric hospitalization, which may inadvertently reinforce the behavior, first responders support a reintegration plan that transitions responsibility back to the PEBE’s existing support system, or initiates referrals to appropriate community-based services. This integrated approach combines the immediacy of CI with the structured behavioral change mechanisms of SBM and ensures both short-term safety and long-term stabilization in a manner that is trauma-informed, scalable, and operationally realistic.

## 4 Concluding remarks

In this chapter, we have introduced an integrated approach that, to our knowledge, has not yet been formally conceptualized. This theory-driven model integrates: (1) observed behaviors and clinically relevant background knowledge as a foundation for decision-making, (2) evidence-based intervention strategies for both mental health crises and maladaptive behaviors below the crisis threshold, and (3) situationally applicable and effective guidelines for first responders. By doing so, we address both the demand and necessity for improved training among first responders in managing PEBEs (e.g., Ghelani, 2022; Lorey and Fegert, 2021; Bock et al., 2015; Richmond and Gibbs, 2021; Thomas and Watson, 2017), as well as the increasing burdens associated with these types of incidents (Krahmer, 2019; Wittmann et al., 2020; Richmond and Gibbs, 2021; Thomas and Watson, 2017).

The iBEAR model provides first responders with a roadmap that can be used not only to respond effectively to emergencies but also to prevent unintended interference with the social structures and supportive routines that caregivers often work hard to establish. For instance, rather than negotiating a compromise that undermines parental authority, such as reversing a screen-time limit during a child’s tantrum, first responders can support existing boundaries and reinforce more adaptive behavioral alternatives. By doing so, they contribute to the preservation of long-term behavioral stability and help sustain the caregiving environment, rather than inadvertently destabilizing it for a quick behavioral emergency resolution. As a result, the iBEAR model will enable first responders not only to de-escalate acute incidents, but also to proactively reduce the recurrence of high-risk behaviors, engage PEBEs more constructively, and promote longer-term stabilization through behavioral shaping and informed, needs-based referrals. Ultimately, the model allows first responders to shape safer, more adaptive responses over time, particularly in cases involving repeat service users, chronic emotional dysregulation, or escalation-prone interpersonal dynamics.

In practical terms, the implementation of the iBEAR model will involve training and educating first responders to recognize and interpret observable behaviors using simplified clinical categories as discussed (suicidal, psychotic, and other behaviors that significantly interfere with the ability to function; section 3.2.1; as well as instrumental suicidal, non-suicidal self-endangering, and other aggressive and disruptive behaviors; section 3.3.1). This will allow them to categorize PEBE presentations either as mental health crisis or maladaptive behavior below crisis threshold, which will then dictate clinically advised and evidence-based courses of action: CI, SBM, or both integrated. This approach provides first responders with a repertoire of core behavioral strategies, which, can be effectively taught in classroom settings and trained through scenario-based instruction. It can be easily and effectively integrated into existing CIT and crisis negotiation training frameworks.

We emphasize that the iBEAR model is a conceptual proposal designed as a pragmatic compromise to enhance field applicability. It simplifies both the behavioral cues exhibited by PEBEs and the corresponding intervention strategies, enabling first responders to apply them in a practical and consistent manner. Unlike many existing frameworks that focus exclusively on acute crises, the iBEAR model expands the scope of intervention by providing structured tools for managing behaviors that fall below the crisis threshold but still present safety risks or service delivery challenges.

As a whole, it has not yet undergone empirical evaluation and will benefit from future research, including both qualitative studies (e.g., interviews with first responders and PEBEs) and quantitative analyses (e.g., randomized controlled field studies in naturalistic settings).

## Footnotes

^1^Using the term first responders, we refer to law enforcement officers (including police, corrections, and other sworn personnel, whose duties include responding to behavioral emergencies) as well as allied (mental) health care professionals (including social workers, psychiatric nurses, and other clinicians), who (co-)respond alongside or together with law enforcement to behavioral emergencies. As mentioned below, a demand of both training and education in behavioral emergency response has been documented by both law enforcement as well as allied mental health care.

^2^We empathically stress that, within the context of the iBEAR model, risk is inherent to crisis intervention not because of the PEBE or any psychiatric condition they may or may not have, but because it occurs within a law enforcement response, typically initiated through 911 or non-emergency calls involving weapons, physical violence, or aggression. In no way do we intend to suggest such risk to be the result of a mental illness itself. Corresponding perceptions are part of a well-documented stigmatization of individuals with mental health disorders (e.g., Wittmann et al., 2020; Schlier and Lincoln, 2014), which has also become prevalent within law enforcement (Morabito and Socia, 2015; Soares and Pinto da Costa, 2019).

^3^The evidence-based and evaluated Integrating Communications, Assessment, and Tactics™ (Police Executive Research Forum, 2016), Module 6 in particular, offers further guidance.

^4^The mental disorders listed as examples are based on the 5th edition of the Diagnostic and Statistical Manual of Mental Disorders (DSM-5; American Psychological Association, 2013) and the 11th edition of the International Statistical Classification of Diseases and Related Health Problems (ICD-11, World Health Organization, 2022).

^5^The focus of this conceptual analysis is on integrating established, evidence-based practices to help first responders effectively manage the diversity and complexity of behavioral emergencies. Rather than offering a comprehensive review of CI and SBM strategies, which would exceed the scope of this contribution, we focus on how these practices can be meaningfully integrated. Accordingly, we provide concise overviews of each approach and reference key sources for readers seeking more detailed guidance on specific response strategies.

^6^Although ABA principles are supported by a broad evidence base, recent literature has documented concerns regarding corresponding interventions and strategies, including general dissatisfaction and reported harm (Anderson, 2023; Leaf et al., 2022; McGill and Robinson, 2020). Additionally, Bottema-Beutel and Crowley (2021) identified widespread undisclosed conflicts of interest within the literature on ABA.

^7^Officer-created or officer-induced jeopardy refers to situations in which officers escalate otherwise containable incidents, often involving PEBEs and no immediate threat to the public, by prematurely initiating contact, resulting in increased risk and potential use of force. For further discussion, see Lee (2021), Smith (2022), and Zaiser et al. (2023).
