# Correction: Beyond one-size-fits-all: managing mental health calls with the integrated Behavioral Emergency Assessment and Response (iBEAR) model

**DOI:** 10.3389/fpsyg.2025.1721766

**Published:** 2025-10-21

**Authors:** Benni Zaiser, Mario S. Staller, Swen Koerner

**Affiliations:** ^1^Independent Researcher, Aurora, ON, Canada; ^2^University of Applied Sciences of Police and Public Administration North Rhine-Westphalia, Gelsenkirchen, Germany; ^3^German Sports University Cologne, Cologne, Germany

**Keywords:** behavioral emergency, person in crisis, crisis intervention, functional analysis, applied behavioral analysis, crisis response, behavioral health crisis, mental health crisis

There was a mistake in Figure 1 as published. The grey text box outlining behaviors associated with “CI” was inadvertently located in the area of “BM” and vice-versa. In addition, figure one states “BM” instead of SBM, as stated in the text. The corrected Figure 1, where the grey text box starting with “suicidal behaviors” is associated with “CI” and the text box starting with “instrumental suicidal behavior” is associated with “SBM”, as well as where “BM” has been substituted with “SBM”, appears below.

**Figure 1:**
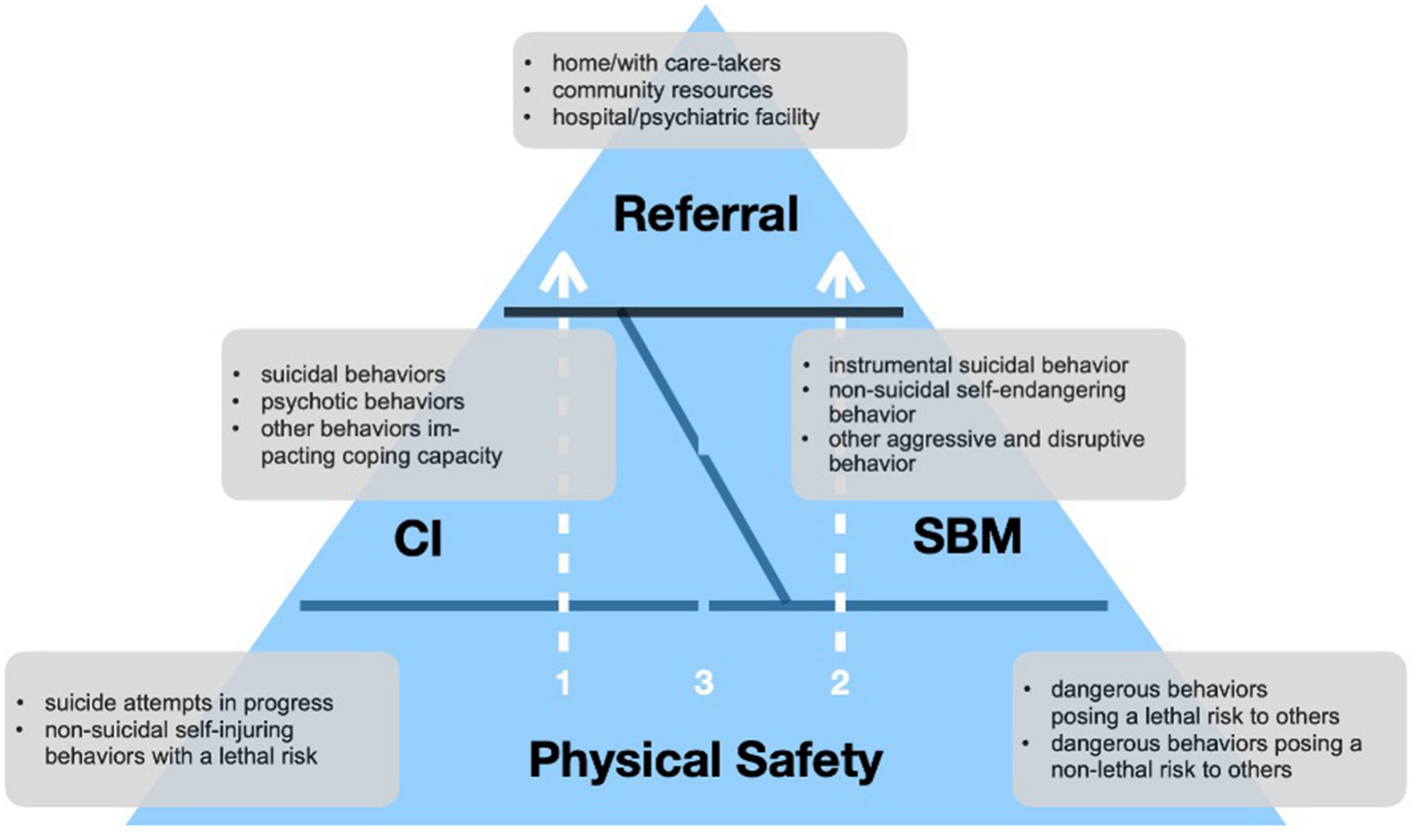
Schematic representation of the iBEAR model.

The original version of this article has been updated.

